# A dual-axis rotation rule for updating the head direction cell reference frame during movement in three dimensions

**DOI:** 10.1152/jn.00501.2017

**Published:** 2017-10-11

**Authors:** Hector J. I. Page, Jonathan J. Wilson, Kate J. Jeffery

**Affiliations:** Institute of Behavioural Neuroscience, Department of Experimental Psychology, Division of Psychology and Language Sciences, University College London, London, United Kingdom

**Keywords:** navigation, three-dimensional space, path integration, spatial cognition, hippocampus

## Abstract

In the mammalian brain, allocentric (Earth-referenced) head direction, called azimuth, is encoded by head direction (HD) cells, which fire according to the facing direction of the animal’s head. On a horizontal surface, rotations of the head around the dorsoventral (D-V) axis, called yaw, correspond to changes in azimuth and elicit appropriate updating of the HD “compass” signal to enable large-scale navigation. However, if the animal moves through three-dimensional (3D) space then there is no longer a simple relationship between yaw rotations and azimuth changes, and so processing of 3D rotations is needed. Construction of a global 3D compass would require complex integration of 3D rotations, and also a large neuronal population, most neurons of which would be silent most of the time since animals rarely sample all available 3D orientations. We propose that, instead, the HD system treats the 3D space as a set of interrelated 2D surfaces. It could do this by updating activity according to both yaw rotations around the D-V axis and rotations of the D-V axis around the gravity-defined vertical axis. We present preliminary data to suggest that this rule operates when rats move between walls of opposing orientations. This dual-axis rule, which we show is straightforward to implement using the classic one-dimensional “attractor” architecture, allows consistent representation of azimuth even in volumetric space and thus may be a general feature of mammalian directional computations even for animals that swim or fly.

**NEW & NOTEWORTHY** Maintaining a sense of direction is complicated when moving in three-dimensional (3D) space. Head direction cells, which update the direction sense based on head rotations, may accommodate 3D movement by processing both rotations of the head around the axis of the animal’s body and rotations of the head/body around gravity. With modeling we show that this dual-axis rule works in principle, and we present preliminary data to support its operation in rats.

## INTRODUCTION

**Fig. 1.**
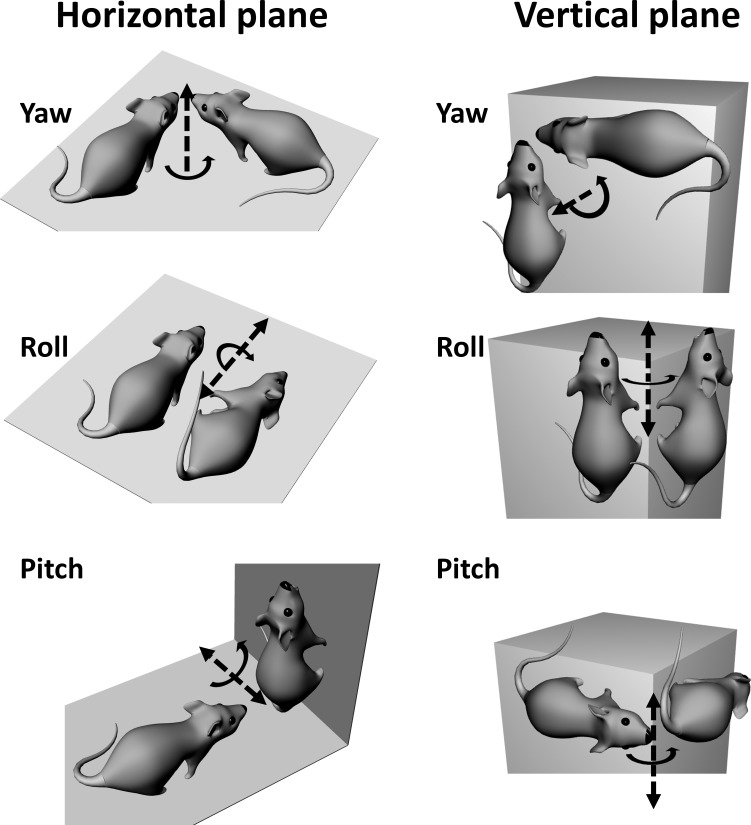
Terminology to describe rotations on horizontal and vertical planes. Axes of rotation are shown by the arrows; dotted lines are the rotation axes and solid lines show the rotation plane. These rotations are described egocentrically (with respect to the animal) but the same terms can also be used allocentrically (with respect to the world). Note that on the vertical plane, the roll and pitch rotations both alter the alignment of the dorsoventral axis of the animal with respect to gravity.

The neural circuitry supporting navigation has been intensely studied for several decades, and attention is now turning to how navigation processes operate in the complex three-dimensional (3D) real world. A core feature of navigation is computation of Earth-referenced head direction, or azimuth, and in mammals this faculty is supported by the head direction (HD) neurons (Taube 2007; Taube et al. 1990a, 1990b), the firing of which is azimuth sensitive. We consider here the problem of maintaining a stable head direction signal when moving over a nonflat surface, which requires rotations in all three cardinal axes (see Fig. 1 for the description and nomenclature of these).

**Fig. 2.**
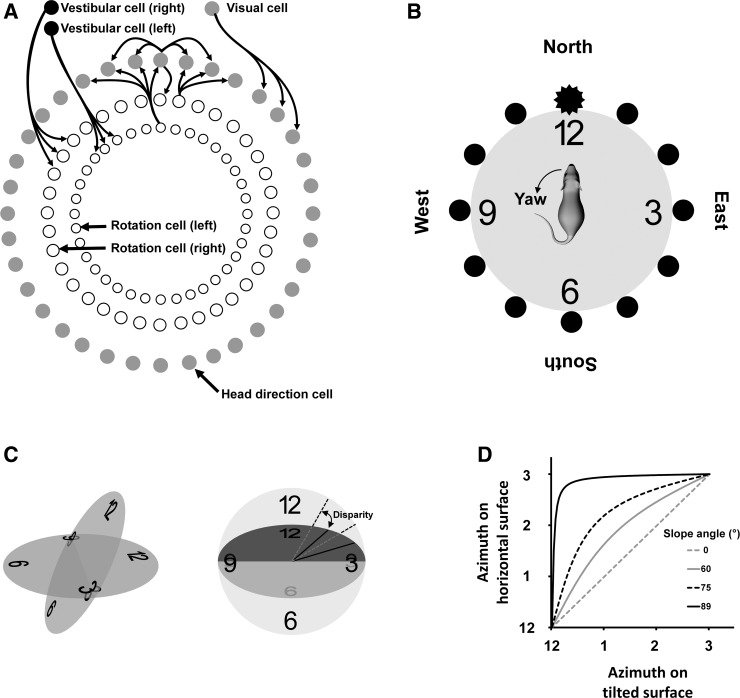
Encoding of azimuth by head direction (HD) cells in two- and three-dimensional environments. *A*: classic organization of a ring attractor, redrawn from Skaggs et al. 1995 with permission. The cells are arranged in a conceptual ring, in which cells activate their neighbors via local excitatory connections. Activity in one part of the ring is initialized by visual input from notional “visual cells.” When the animal turns its head in a yaw rotation, the combination of sensory inputs, mainly vestibular, that signal the change activate “rotation cells” that cause activity to move in the appropriate direction around the ring, at the appropriate rate, so as to keep the representation concordant with real head direction. *B*: schematic of the hypothetical ring organization of HD cells, relative to their two relevant reference frames: global and network. Connections between neurons (see *A*) have been omitted for clarity. The cells (black circles) have, for illustration, been assigned positions on a clock face (numbers) that indicate their positions within the attractor network; the “clock” as a whole is aligned (using landmarks) with the outside world, such that activity of the “12” cell (starred) corresponds to the animal facing North. When the animal makes a yaw turn of its head, activity moves around the attractor ring at the same rate as the head rotates in the actual world, to keep the internal directional representation aligned with real heading. *C*: the relationship between yaw and azimuth becomes altered when the animal is no longer on a horizontal plane. Shown here are the effects on azimuth calculations when a horizontal surface is compared with the same surface tilted by 60° (*left* view). The lines (*right* view) show the notional 1 o’clock and 2 o’clock cells, dotted for the horizontal angular positions and solid for the tilted ones. Note the mismatches: e.g., 1 o’clock on the tilted surface maps to ~2 o’clock on the horizontal. *D*: quantification of the effect illustrated in *C*, showing how the mismatch between positions varies as a function of the amount of tilt. Thus, any computation that tries to extract azimuth from the clockface ring attractor needs to adjust not only for yaw rotations but also for the amount by which the surface is tilted.

HD neurons are thought to be organized in a so-called ring attractor (Calton et al. 2008; Knierim and Zhang 2012; Skaggs et al. 1995; Zhang 1996; Fig. 2*A*), in which active neurons suppress activity elsewhere in the circuit, such that only one direction (the facing direction) is signaled at any one time. The activity of the neurons is updated by changes in head direction (i.e., yaw): a simple “transfer function” describes the movement of activity through the HD cell network so that a given head turn moves activity around the ring attractor by the appropriate amount. This means that HD cell activity always reflects the animal’s current azimuth: on a horizontal surface, yaw thus corresponds linearly to azimuth change.

**Fig. 3.**
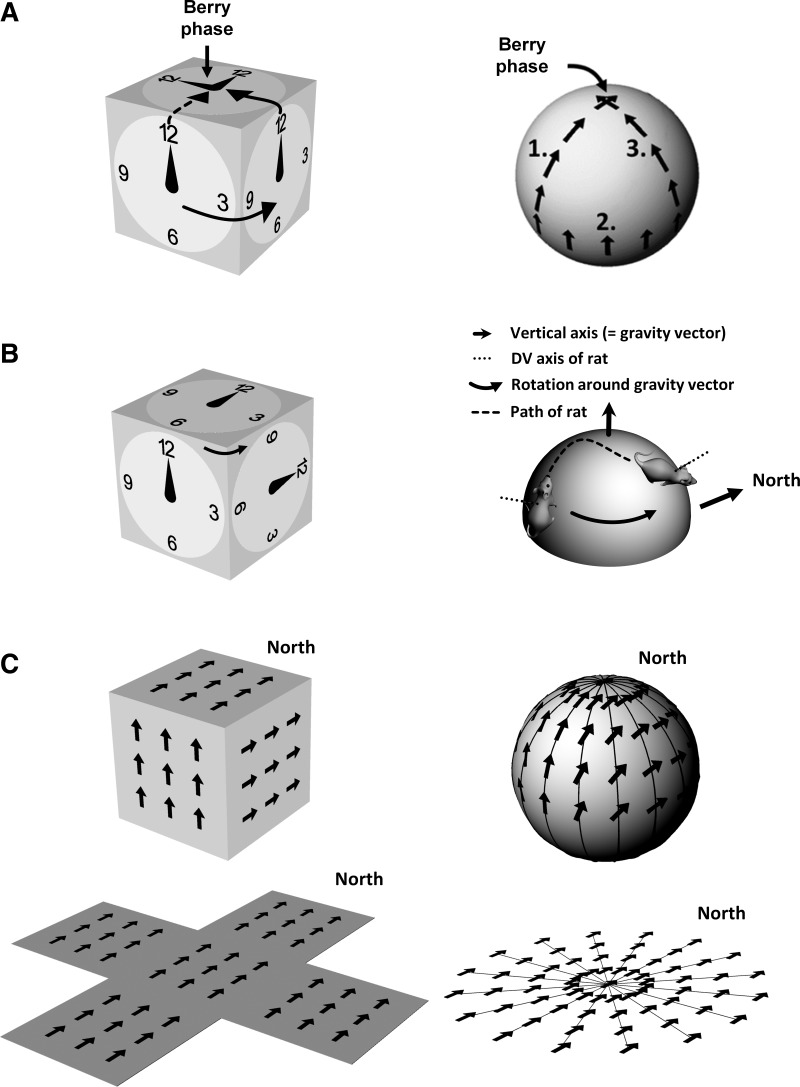
Correction of HD cell errors following movement over nonhorizontal surfaces. *A*: the acquisition of error (“Berry phase”) in a hypothetical HD cell that is insensitive to rotations of the locomotor surface. The *left* plot shows a simplified environment having orthogonal surfaces (a cuboid); the *right* shows the same effect on a sphere. On the cuboid, the directional firing preference of the HD cell is shown by the hand on the clock face, while the alignment of the entire HD cell ring attractor is shown by the clock face itself. If the system is insensitive to rotations of the locomotor surface around the vertical (gravity-aligned) axis then the “12” cell fires when the animal faces up on all the vertical surfaces. On the top surface, both alignment of the ring attractor and the firing of the HD cell are different depending on which surface the animal had traveled from — this difference is the Berry phase error. The *right* plot was taken from Jeffery et al. (2015) and shows Berry phase error for a HD cell transported over the surface of a sphere. The principle is the same: an error accrues on the top surface following a three-step journey (shown by the numbers 1–3) over the sphere’s curved surface. *B*: correction of the directional alignment of HD cells by rotations linked to the rotation of the locomotor surface. The *left* plot shows adjustment of the HD cell ring attractor (the clock face) following movement from one vertical surface to another; this adjustment means that firing on all surfaces is congruent, and no Berry phase error accrues. The *right* plot shows generalization of the rotation rule to a sphere. The rotation of the locomotor surface is detected by detecting the rotation of the rat’s dorsoventral (D-V) axis around gravity, at each time point as it moves over the sphere’s surface. *C*: correction of Berry phase errors by rotating the signal to account for surface rotation means that the signal essentially points in the same direction (e.g., “North”) everywhere on the surface. This can be seen by “unfolding” the surface to make it flat, and is true both for the cuboid (*left*) and sphere (*right*). Another way to think about it is that the signal adopts the closest angle to North that it can get, given the constraints imposed by the surface orientation.

Computing a stable azimuth signal during movement over a nonhorizontal surface, however, poses challenges (Calton et al. 2008; Clark et al. 2012; Finkelstein et al. 2015; Stackman et al. 2000; Taube et al. 2004, 2013; see Jeffery et al. 2015 for discussion). Three important problems are as follows:

There are slope-dependent nonlinearities in the transfer function describing the shift of activity through the network (Fig. 2). This is because the relationship between a yaw head turn and change in azimuth depends on the steepness of the slope the animal is on (Fig. 2*B*), becoming increasingly nonlinear as the steepness increases (Fig. 2*C*). The transfer function, however this is implemented in neurons, thus needs to be slope sensitive.
Errors in azimuth computation, known as Berry phase, occur following movement over differently oriented nonhorizontal surfaces (Fig. 3). This happens because when the surface changes direction (e.g., as the animal rounds a vertical corner), the reference frame of the directional signal changes its relationship to the world at large. This is shown in the figure, where the reference frame is depicted by the clock face. The “12” HD cell, which fires (indicated by the hand on the clock) when the animal faces upward, does so on all vertical surfaces but its direction in allocentric space changes — this can be seen by transporting the animal back to the top surface from either of the two visible vertical surfaces and observing that the cell’s resultant azimuthal firing direction is different. The direction of firing on the top surface after an excursion over nonhorizontal surfaces is thus path dependent, which is obviously not useful for spatial orientation. This is also true for movement over a sphere, and by extension any undulating surface, even if the surface orientation varies continuously instead of suddenly.
If, instead of simply encoding azimuth, the brain constructs a fully 3D compass, with cells for every heading direction in volumetric space, then there are capacity issues. Such an encoding would need vastly more neurons, most of which would be silent most of the time since animals rarely point in all possible directions during the course of a day. Since neurons are extremely expensive, in resource terms, to construct and maintain, this would be highly nonoptimal if a simpler work-around could be found.

One such work-around would be to adapt a horizontal compass for use in 3D, simply by adding a correction factor to remove Berry phase errors following rotations that are not on the horizontal plane. The correction amounts to detecting how much the surface has rotated with respect to the gravity-defined vertical axis and then adjusting the HD signal by that amount. This correction is shown in Fig. 3, *B* and *C*, illustrating a HD cell that is “trying” to point North. On a cube, if the animal rounds a vertical corner then the entire HD cell reference frame — the clock face — is rotated by the same amount (90° in this case) so that activity of the HD network updates by 90°. When the animal climbs back to the top from either surface, since it now is not making a vertical rotation, no additional adjustment is needed. Firing on the top surface is thus congruent with all of the vertical surfaces (as shown in Fig. 3*C* where the cube has been unfolded). The rule generalizes to a sphere — as the rat moves over the surface, the rotation of the sphere’s surface, determined by the slight rotation of the animal’s dorsoventral (D-V) axis at each time point, is also applied to the HD signal so that the orientation of the HD network is adjusted continuously as the rat traverses the sphere surface — again, this means that firing everywhere is congruent (with the exception of the undersurface of the environments, which we consider separately later on). The firing direction of a North cell is, on a nonhorizontal surface, as close to North as it can get, and the animal can thus remain oriented in allocentric 3D space.

**Fig. 4.**
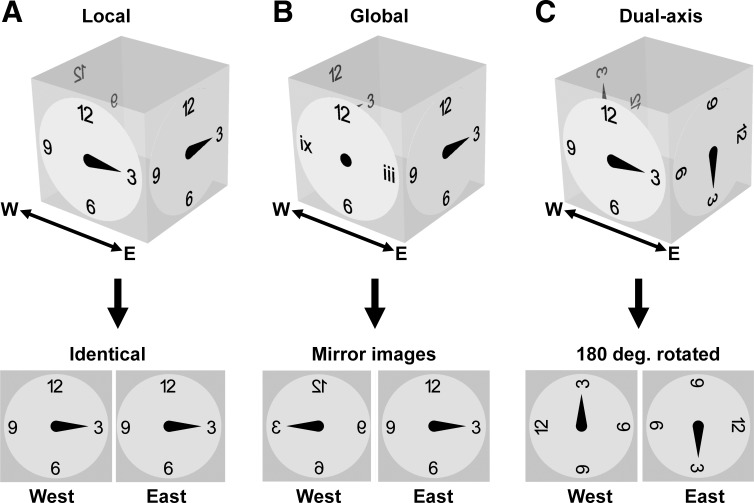
Prediction for HD cells on opposing “East” and “West” surfaces with and without detection of surface orientation changes. *A*: for a purely local rule, HD cells are sensitive to yaw only and insensitive to surface orientation changes. Firing on opposing surfaces will thus have the same local pattern. *B*: for a global HD system in which every direction in 3D space has a unique cell representation, then the pattern of firing on the two walls will form mirror images, and a different set of cells will be active on orthogonal surfaces except for the common directions of “up” and “down.” Here we have used Arabic numerals to denote the reference frame for activity in one of the dimensions (the two walls having no component in the East-West dimension) and Roman numerals for activity in the second dimension (the walls with no component in the North-South dimension). The third dimension, the two horizontal surfaces, is not annotated here. *C*: with a dual-axis rule, rotations around corners are detected and used to update the HD cells; since there are two 90° rotations between East and West walls, this means the pattern will rotate by 180° between the walls.

There is experimental evidence that HD cells indeed maintain a planar representation even on a vertical surface. Stackman et al. (2000) found that HD cells would continue to fire in unchanged fashion when a rat moved from a floor to a wall, as if the system was insensitive to the pitch rotation. In a follow-up experiment they showed that, while on the wall, firing continued to be updated in the usual way following yaw rotations (Taube et al. 2013). Calton and Taube (2005) also found, as discussed later, that firing during complete inversion became nondirectional, pointing to a limitation in the capacity of the system to track movement in 3D space. However, these studies did not investigate whether firing directions would rotate when the animal rounded a vertical corner, rather than climbing onto a vertical wall from a horizontal surface. In the present study we modeled the rotation proposal using a ring attractor, to test its feasibility and robustness, and then conducted a HD recording experiment to look for updating of the HD signal as a function of turning vertical corners. We tested the rule against the two other possible coding schemes for HD cells: a purely local one in which surface rotations are not compensated for, and a fully global one in which every direction in 3D space has a unique cell assignment. These are shown in Fig. 4, *A*–*C*.

The three schemes make three predictions for how HD cells should fire on opposing walls:

The local scheme (Fig. 4*A*) is the one that would operate if there was no active rotation of the HD signal as the animal rounded a vertical corner. It predicts that within a global (room) frame of reference, each of the firing patterns on two opposing surfaces would each be a 180° rotation of the other, while the patterns as seen from the perspective of the two facing cameras would be identical.
The global scheme (Fig. 4*B*) is the one that would operate if the HD compass is fully 3D. It predicts that on opposing walls, firing would maintain a consistent direction relative to 3D space, while the patterns as seen from the two facing cameras would be mirror images. This model was proposed, but then discounted, by Finkelstein et al. as a possible way to describe the HD firing patterns seen in bats (Finkelstein et al. 2015).
The dual-axis scheme (Fig. 4*C*) is the one we proposed above, in which the signal is rotated according to vertical turns as well as by yaw turns of the head. It predicts that within the global reference frame firing would be flipped vertically, while the patterns seen from the camera views would be rotated (rather than mirrored) with respect to one another. This alternative resembles the local hypothesis in that it preserves the same relative firing angles between neurons, but differs in that it allows firing to maintain consistency with the global (room) reference frame too. This scheme allows for a mosaic-like representation of 3D space, composed of multiple 2D fragments, and thus has also been called a "mosaic model" (Dumont et al. 2017; Wilson et al. 2016). Using this scheme, a rat could climb onto the roof from either East or West walls without inducing HD cell conflict (Fig. 3*A*).

## EXPERIMENT 1: MODELING

### 

#### Methods.

Details of the model are outlined in the appendix; a synopsis is presented here.

We modeled the dual-axis rule in a simulated ring attractor, to check that it works as predicted and generalizes to arbitrary surfaces. The model is based on a Continuous Attractor Neural Network (CANN), a well-established architecture for modeling the head direction system (Knierim and Zhang 2012; Skaggs et al. 1995; Stringer et al. 2002). A ring of HD cells is topologically organized by preferred firing direction, plus a Gaussian-distributed activity focus (“packet”) within the network to represent current head direction. Cells are connected by prewired fixed-weight recurrent collateral synapses, with the strength of each connection depending on the similarity of preferred firing directions. Individual cells are modeled as leaky-integrator firing rate neurons in which activity is represented as an instantaneous average firing rate for each cell, with an axonal delay between one cell and the next.

According to the dual-axis rule, the current estimated HD of the animal, HD(*t*) is updated based on previous HD, HD(*t*−1), the rotation of the head around the D-V axis (*I*^AHV^), and rotation of the D-V axis around the vertical axis defined by gravity (*I*^G^)

$$HD\left(t\right)=HD\left(t-1\right)+I^{AHV}\left(t\right)+I^{G}\left(t\right)$$

To implement the rule, all HD cells receive a Gaussian path integration input, centered on a point determined as the previous ring location to which input was applied at the previous timestep together with an increment based on the combined amount of D-V-axis and gravity-vector rotations that has occurred between *t*−1 and *t*. If no change occurs, there is no external input.

Custom-built MATLAB scripts were used to generate a random walk over the surface of either a cuboid, a hemisphere, or an inverted hemisphere referred to as a “bowl” (the cuboid notionally having dimensions identical to the tree-trunk apparatus used in the experiment; namely 50 × 50 × 80 cm; the hemisphere and bowl having radius 50 cm). Every 100 ms in model time the simulated rat took a step of length 2.5 cm, the direction of which was selected from a Gaussian probability distribution centered ahead of the rat.

Trials were 600 s long, generating 6,000 pairs of position points, each pair corresponding to the beginning and end of a step. If a rotation about the D-V axis, or of the D-V axis about the vertical axis, occurred during a step, the head direction firing packet location was updated according to the dual axis rule.

Details of model analysis are again given in the appendix, and a synopsis is presented here for easier understanding.

To analyze model performance at a network level, a population vector statistic was taken to determine the location of HD activity in 360° space. At each model timestep, the rat was rotated back to the earth horizontal via the shortest distance. This was done by aligning the D-V axis with the vertical axis defined by gravity [i.e., the *z*-axis (0,0,1)]. The difference between the heading of the rat when rotated back to the earth horizontal was compared with the HD population vector and expressed as the new preferred firing direction of the HD cell that preferred North (0°) at the start of simulation.

For the cuboid (tree-trunk) apparatus, tuning curves were calculated by recoding HD into the frame of reference for the current wall (North, South, East, West) and binning in 6° increments. The tuning curve of a given HD cell was taken as the mean firing rate in each bin.

#### Results.

**Fig. 5.**
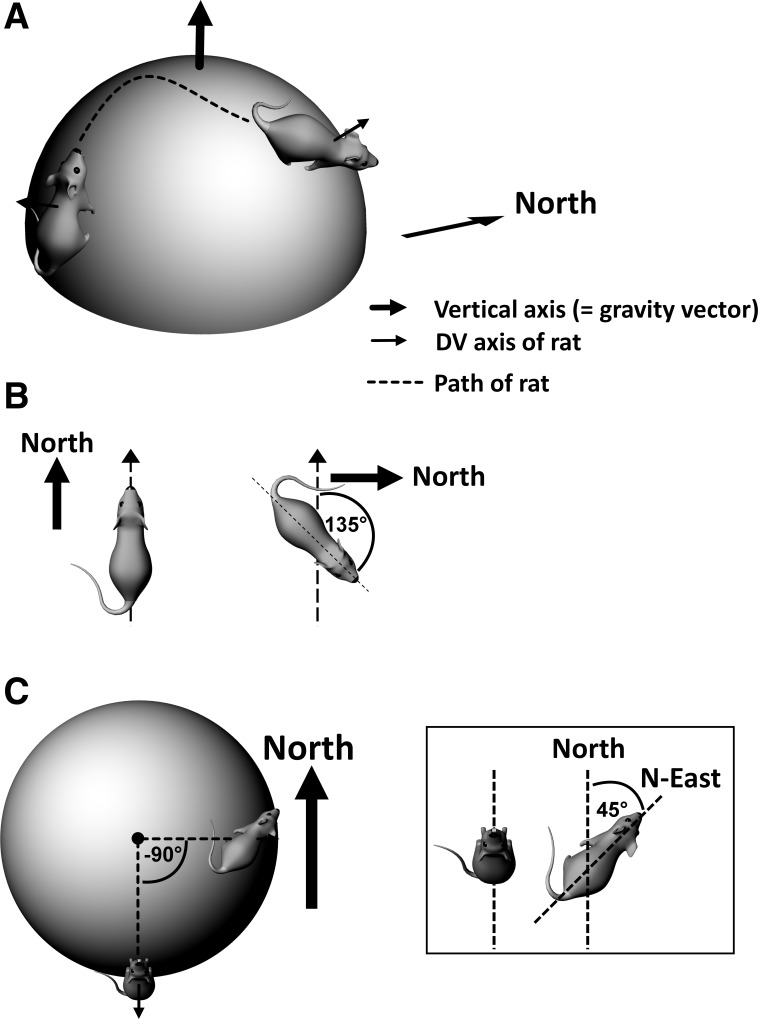
HD cell updating as an animal traverses a curved surface. *A*: a rat follows a path (dotted line) from one point to another over the surface of a sphere. The dorsoventral (D-V) axis of the rat, orthogonal to the locomotor surface (the sphere tangent plane at that point) is shown by the small solid arrow. Note that between the start and end of the journey segment, this axis has rotated with respect to gravity. *B*: the rotation of the rat around its D-V axis. In each case the viewpoint is directly above the animal. Between start and end condition the rat rotated 135° clockwise (CW) in its own, egocentric reference frame. However, because it also walked over the surface of the sphere, it gained a rotation in the other direction, too, so its rotation with respect to North is only 45°. *C*: the same situation now viewed from directly overhead. Because of the change in the orientation of the surface the animal is standing on, the rat’s D-V axis has rotated around the vertical axis (or gravity vector) by 90° counterclockwise (CCW). Together with the 135° yaw rotation it made, this accounts for the 45°CW rotation with respect to North. *Inset*: the head direction system should thus add the local rotation of the rat (135°CW) and the rotation of the surface (90°CCW) to get a reading of true allocentric head direction of 45° East of North.

**Fig. 6.**
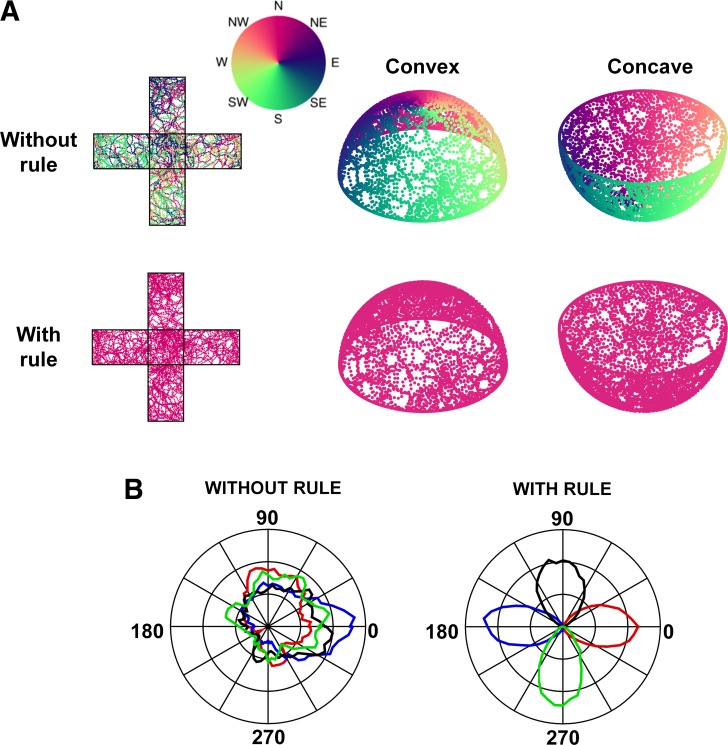
Simulating the effect of the gravity-vector rule on the consistency of head direction cell firing on three-dimensional surfaces. *A*: the two *leftmost* panels illustrate the pattern as seen from above on the unfolded cuboid apparatus, with or without the dual-axis gravity rule operating. Positions of a simulated exploring rat were sampled every 100 ms: each dot represents the current state of the North head direction cell at that point, colored according to the direction it would be firing (see color map) if the simulated rat were to move directly (i.e., without incurring any yaw rotation or rotation of the D-V axis) to the uppermost, horizontal surface of the apparatus. Without operation of the gravity-based dual-axis rule, firing would be inconsistent, as shown by the variable colors on each surface. With the rule, the North cell would always fire when the rat faced North. The *middle* two and *rightmost* two panels show the same simulation run when the simulated rat explored a convex (*middle*) or concave (*right*) hemisphere (“dome” and “bowl,” respectively). The difference in error pattern between the cuboid and hemispheric apparati (intermingled errors for the cuboid, smoothly transitioning errors for the hemispheres) is due to the fact that the errors that accumulate for rotations about gravity in one direction are corrected again by rotations in the other direction, except for the special case when the rat moves to the top of the apparatus directly without rotation. This rarely if ever happens on the hemispheres and always happens on the cuboid. *B*: polar plots showing firing profile of network cell 250 in the camera frame of reference for each wall for model-generated data on the simulated tree-trunk maze with (*right*) and without (*left*) the dual-axis rule. Firing rate data were averaged across 6° bins of directional heading within the current wall. Data for the East wall is plotted in blue, the West wall in red, the North wall in black, and the South wall in green. Without operation of the dual-axis rule (*left*), there is no consistent relationship between cell firing and a given head direction within the current wall. With the dual-axis rule (*right*), firing is strongly related to head direction within the current wall. Preferred firing directions on opposing walls are separated by 180°. Additionally, preferred firing directions on adjacent walls are separated by 90°.

The dual-axis rule is graphically depicted in Fig. 5 for an imaginary rat walking on a hemisphere, showing how the head turn and the D-V axis turn are extracted for each timestep and used to update the HD signal. Figure 6*A* shows the activity of the head direction network after exploration over either a cubic or hemispheric surface (convex — “dome” or concave — “bowl”). Figure 6*B* shows the activity of an example cell from the network. Using only a local rule, HD cell firing progressively deviates in both of the 3D environments. On the tree trunk, errors occur abruptly when the rat crosses a corner, eliciting a sudden rotation around the vertical axis. On the hemisphere the errors accumulate gradually, because each step rotates the animal incrementally around the vertical axis (except in the occasional cases where the animal heads straight toward the upper pole). In the presence of the dual-axis rule, by contrast, activity is always correct in both environments. Note that the rule would work for both convex surfaces, when the D-V axis slopes away from the vertical axis, and concave surfaces where it slopes toward it as demonstrated by the bowl simulations.

Figure 6*B* shows model behavior at the single-cell level for an example cell. In the absence of the dual-axis rule, cell firing does not have a consistent relationship to head direction in the current wall's camera reference frame. In contrast, during simulations incorporating the dual-axis rule, the cell shows a clear preferred firing direction on a given wall, with transitions between walls causing a 90° rotation of preferred firing direction.

## EXPERIMENT 2: ELECTROPHYSIOLOGICAL RECORDING

The experiment aimed to test the model predictions by recording HD cells as rats moved between oppositely oriented vertical surfaces.

### 

#### Subjects.

Subjects were three adult male Lister Hooded rats, weighing 300–350 g at the time of surgery. All procedures were licensed by the UK Home Office subject to the restrictions and provisions contained in the Animals (Scientific Procedures) Act 1986. From weaning age (21 days) until surgical electrode implantation the rats were raised in littermate groups of four to eight in a large parrot cage (Rainforest Cages, Sky Pet Products, Northamptonshire, UK), 2.2 m high and 2.2 × 1.5 m width and depth. The cage was lined with chicken wire and filled with climbing structures, which the animals spent most of their time traversing: by the time of recording they were therefore highly skilled at climbing and would have had ample opportunity to form a full, 3D spatial representational competence.

After surgery they were individually housed in standard cages to prevent their damaging each others’ implants. Lighting was set to a reversed light-dark cycle, with simulated hour-long dawn and dusk periods at 11 PM and 11 AM, respectively. After a 7-day postsurgery recovery period the animals were placed on a restricted diet sufficient to maintain 90% of their free-feeding weight.

#### Apparatus.

For initial identification of HD cells, screening took place in a 70 × 70 cm square box with 50-cm-high walls, located in the center of a standard laboratory room and adorned with a cue card on one wall, to aid the animal’s orientation. An overhead camera monitored the position and heading of the animals by detecting two LEDs on the implant headstage.

**Fig. 7.**
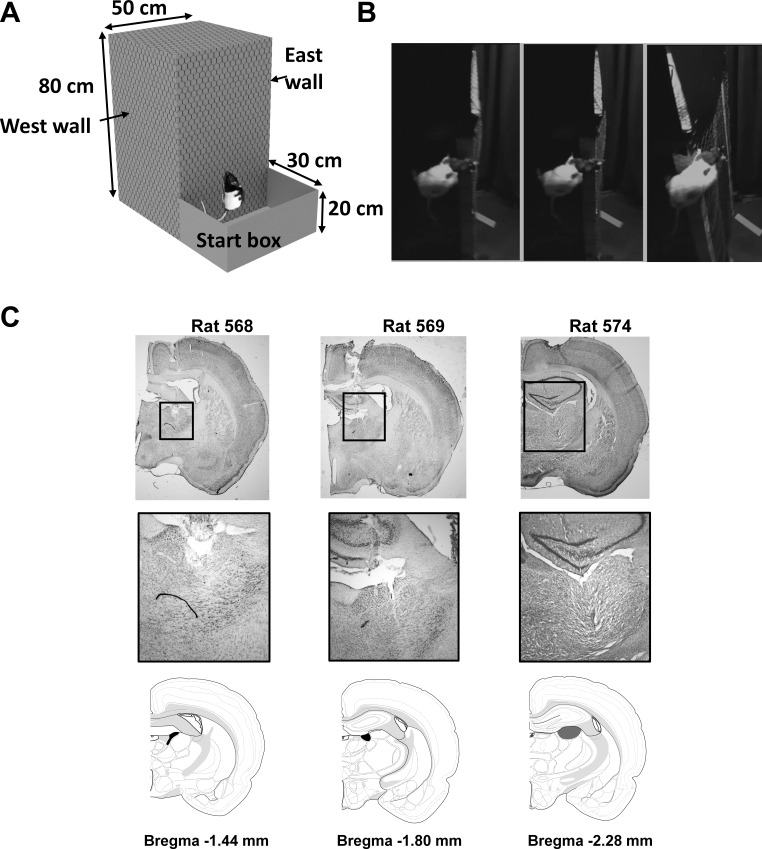
Experimental apparatus and histology. *A*: schematic of the structure of the tree-trunk maze. *B*: video stills of the rat circumnavigating a corner, using the typical pose which is to pitch around the mediolateral body axis, which rotates the D-V axis 90°. with respect to gravity. *C*: coronal thionin-stained sections from each of the three rats showing electrode tracks. Arrowheads on the photomicrographs show the estimated final electrode location, which are then mapped to the line drawings below, which are taken from Swanson (2004), maps 25, 27, and 28 with anterior thalamus filled in black. HD neurons were generally estimated to be in anterior thalamic nuclei and were assumed to be in the dorsal part, although as electrode tracks extend beyond the recording zone we cannot rule out that a small number may have been more ventral, where HD neurons have also been reported (Tsanov et al. 2011), or for rat 574 more lateral, in lateral dorsal thalamus (shaded in gray) where HD cells have also been reported (Mizumori and Williams 1993).

The experimental apparatus (Fig. 7*A*), which we named the “tree-trunk maze” because it mimicked a naturalistic navigational setting, was located in the center of a standard 3 m × 2 m laboratory room and was surrounded by a 2-m-diameter circular array of floor-ceiling black curtains, so as to reduce the availability of extramaze cues. The apparatus itself was an oblong structure 80 cm in height and 50 × 50 cm in width and depth, covered in chicken wire to aid climbing. Further 30-cm-high smooth wall-extensions were attached to the top of each of the sides of the climbing apparatus to prevent the animals from climbing on to the top surface of the apparatus. Attached to the bottom of one wall (defined arbitrarily as “South”) of the climbing structure was a starting box, measuring 50 × 30 cm and with 20-cm-high walls on the three exposed sides, from which rats would start all trials. Two cameras were used to track position and heading on the opposing East and West walls. Their signal was passed through a time-base corrector (Datavideo TBC-5000) before being relayed to the DacqUSB system unit, alternating frames between the two cameras at 50 Hz (each camera thus being relayed at 25 Hz).

In all climbing trials rats climbed onto the South wall from the starting box before then climbing around onto one of the two recording walls. On returning from the recording wall rats then climbed back around to the South wall before returning to the starting box. Throughout training and experimental sessions rats were encouraged to return to the start box for rest periods between the East and West climbing epochs, to maximize sampling time on the walls. Rats on average carried out a total of 5.9 ± 0.7 climbs on the West wall and 5.5 ± 0.6 climbs on the East wall, in each session.

Rats were rewarded with a malt paste food reward that was dotted around on the two recording walls. A small amount of the food paste was also presented to the animal upon return to the starting box. Further reward was given to the animal on the wall using a cotton bud applied by the experimenter; this was done to encourage yaw rotations beyond ±90°.

#### Presurgery training.

All rats received at least 5 days of presurgical training on the tree-trunk maze. This training included 2 days of 15-min-long habituation to the starting box. Following these 2 days rats were encouraged to climb onto the South wall of the structure to receive food reward, and once they were willing to do this regularly were encouraged to climb around to the East and West walls of the apparatus for further reward. This was repeated for at least 3 days, or until rats willingly climbed from the starting box to the South wall, and around to the East and West walls with ease.

#### Electrodes and surgery.

After presurgery training, the rats were implanted with moveable 17-μm-diameter platinum-iridium (H-ML insulated) microelectrodes (California Fine Wire), configured as either single electrodes (two rats) or four twisted-quadruple tetrodes (one rat) and carried by 16-channel microdrives (Axona, St. Albans, UK). Electrodes were implanted just above the anterodorsal nucleus of the thalamus (ADN) at coordinates 1.8 mm posterior from bregma, 1.4 mm lateral to the midline and 2.1 mm ventral to the brain surface. After surgery the rats were given meloxicam (Metacam, 0.2 mg/kg). All animals were given at least 1 wk to recover from surgery before screening commenced.

#### Screening and recording procedures.

Single-unit recording was carried out using a multichannel DacqUSB recording system (Axona). To record units, animals were connected to a preamplifier via a lightweight cable attached to the microdrive by a headstage that modified the signal with AC-coupled, unity gain operational amplifiers. The signal was amplified ~15,000 times and bandpass filtered between 500 Hz and 7 kHz. Recording thresholds were set to ~70% above baseline activity levels, and data from spikes above the threshold from all channels were collected across a period spanning 200 µs preceding the peak amplitude of a spike and 800 µs following the peak amplitude. The activity of channels from any given tetrode was referenced against the activity of a single channel from another tetrode.

Beginning at least 1 wk after electrode implantation, screening for HD cells took place. During screening sessions, the position and head direction of the animal were recorded using an overhead camera. Two LEDs of different sizes were mounted on the headstage with a separation of 5 cm. The position of the animal in the environment was estimated as the average pixel position in *x* and *y* coordinates between the two LEDs, and the animal’s heading direction was determined using the two LEDs. During screening, the digital oscilloscope was monitored for spiking activity; if this was detected then a baseline recording session, of at least 5 min, would follow, during which animals were encouraged to move through the environment to forage for food rewards of either cereal, rice, or condensed milk droplets. If no HD cell activity was present (methods described in *Data analysis* below), the electrodes would be lowered by ~50 µm. If HD cells were found, the animals would progress onto the experimental protocol on the tree-trunk maze.

For the experimental protocol, recordings were run continuously in sessions of up to 20 min, although sometimes multiple sessions were required to ensure adequate directional coverage on the walls. At the start of each recording session the rat was placed in the starting box of the apparatus and would then climb onto the South wall, before climbing around under its own volition to either the East or West walls to collect food reward. At times, the rat was encouraged with a small trail of food to climb to one particular wall to increase the sampling of that wall. Once on the recording walls, the rat was also further encouraged to carry out yaw rotations using food reward placed on the end of a cotton bud, to increase sampling of downward facing head directions, which the rats seldom sampled without encouragement.

#### Data analysis.

Because climbing sessions were run using continuous recording, it was necessary to isolate the climbing epochs seen by each camera and then to concatenate these to produce a composite trial for each wall. This was done by stepping through the path of the animal as recorded by each camera and splitting the recorded file into climbing epochs, each beginning and ending with the entry and exit of the rat onto the wall. The first and last 2 s of each epoch were excluded to prevent the data being contaminated by the intermediate period when the corner was being negotiated. The heading direction of animals was computed using the two LEDs on the headstage and was grouped into 64 directional bins.

We analyzed data on a per-session basis rather than a per-rat basis, averaging results where more than one cell was recorded in a session. The electrophysiological data for each wall was analyzed separately, with predefined HD cells identified using Tint cluster cutting software, before being analyzed in MATLAB (MathWorks, Natick, MA). Cluster cutting was done by hand, as clusters were generally well separated. Once single cells had been isolated, the heading direction of animals was correlated to the neural spiking activity of each cell.

Having extracted the directional and positional data plus the spikes for each cell for each trial (baseline, West wall, East wall), tuning curves were generated and from these, basic firing parameters were calculated. Tuning curves were constructed by dividing the number of spikes in each directional bin by the dwell time, and then Gaussian smoothing using a kernel of 5°. Basic firing parameters were *1*) peak and mean firing rates, *2*) Rayleigh vector lengths, *3*) angular standard deviations, and *4*) directional difference in firing between two trials. These analyses are detailed below. Cells were considered to be directional if they had a peak firing rate over 1 Hz, exhibited more than 100 spikes during screening session, and had a significant mean resultant vector.

The peak rate was defined as the firing rate in the bin with the highest value; the mean rate was the sum of the rates in all the bins divided by the number of bins.

The mean resultant vector length, otherwise known as the Rayleigh vector, was calculated as

$$r=\sqrt{x^{2}+y^{2}}$$

where *x* and *y* are the rectangular coordinates of the mean angle. Rayleigh vector values range from 0 to 1, with values closer to 0 indicating that the cell activity is not directionally modulated and fires equally in all directions, and values closer to 1 indicating directionally modulated cells. *Z*-scores and their associated significance values could then be calculated to determine whether the firing of a given cell was significantly directional, using the following equation (where *n* is the sample size):

$$z=nr^{2}$$

The directional difference in firing between a pair of plots was determined using rotational cross-correlations, in which one plot was rotated against the other in 1-bin steps, with Pearson’s *R* recalculated at each step. After the full 360° set of cross-correlation computations, the offset with the highest positive correlation was taken as the rotational difference between the two trials.

Model predictions were derived by taking the West wall data and then either keeping unchanged (local rule, Fig. 4*A*), reflecting in the vertical axis (global rule, Fig. 4*B*) or rotating by 180° (dual-axis rule, Fig. 4*C*). The recorded directions were then subtracted from the predicted directions to yield the model prediction error.

#### Histological analysis.

At the end of experiments, which was usually determined at the point at which electrodes were considered to have moved beyond the region of interest, or when HD cells had not been observed for a matter of weeks, animals were euthanized and perfused with paraformaldehyde. Brains were sliced at 40-μm thickness coronally and stained with thionin solution.

#### Results.

**Fig. 8.**
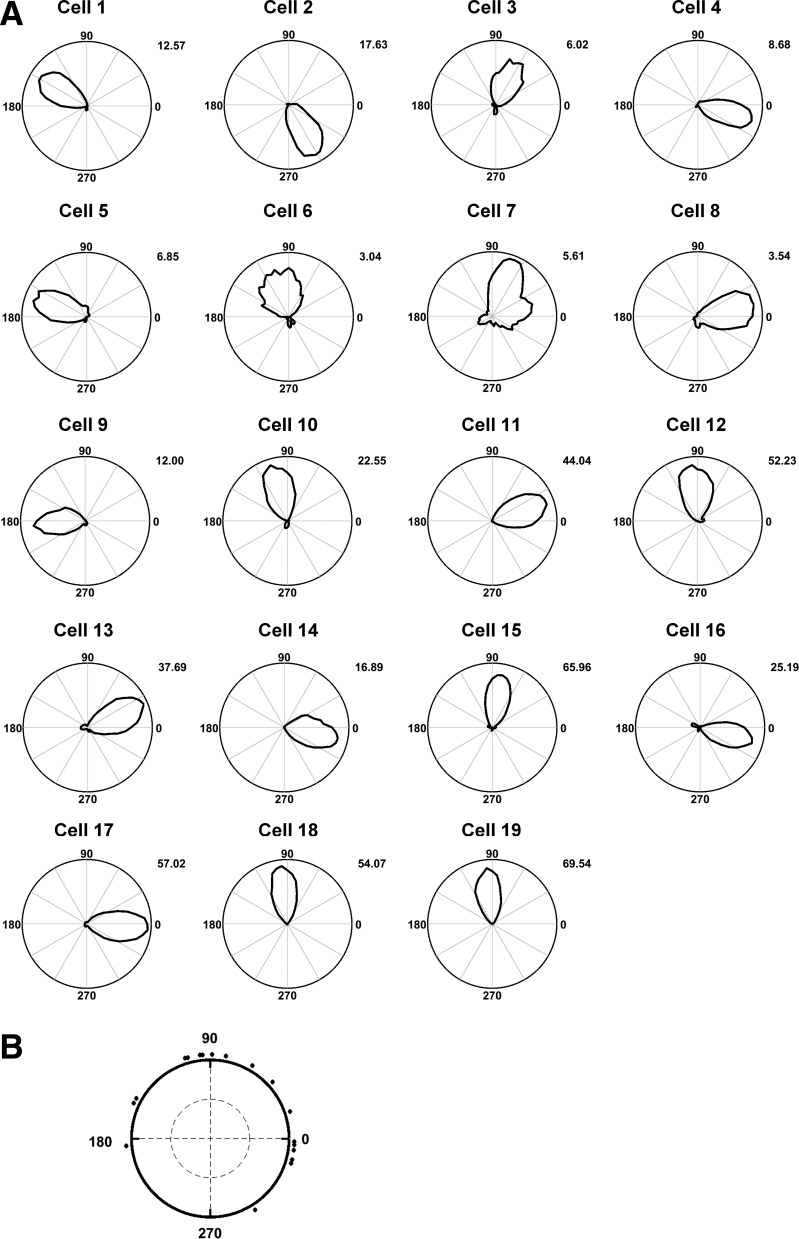
*A*: head direction cell tuning-curve polar plots for the complete set of recorded neurons, during the baseline trial. Cells 10, 14, and 17 were from rat 568; cells 18 and 19 from 574; and the remainder from rat 569. Peak firing rates (Hz) are shown for each plot. *B*: mean firing direction of all cells during the baseline trial.

**Fig. 9.**
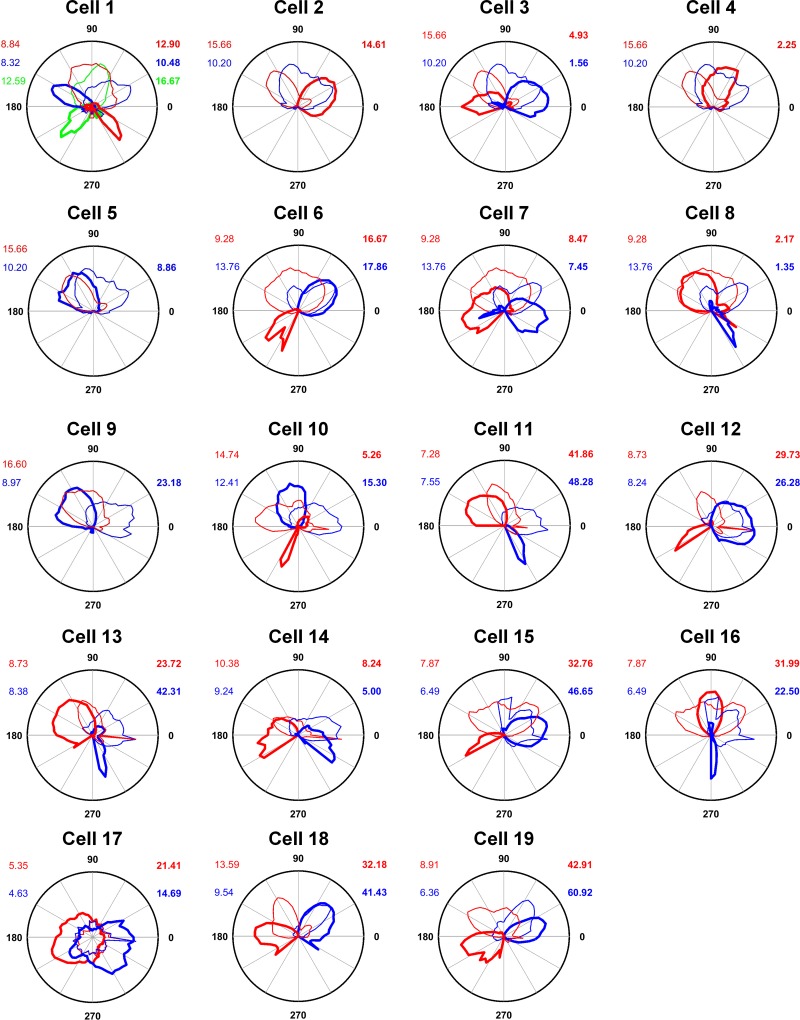
Head direction cell tuning-curve polar plots for the complete set of recorded neurons, on the East (blue) and West (red) walls. Cell 1 was also recorded on the South wall (green). Cells 10, 14, and 17 were from rat 568, cells 18 and 19 from 574, and the remainder from rat 569. 0 = right; 90 = up; 180 = left; 270 = down. Peak firing rates (Hz) are shown in corresponding bold colors, and peak dwell times in corresponding nonbold colors. Cells 2, 4, 5, and 9 had below-threshold firing on one wall, likely due to inadequate directional sampling in the direction opposite to the existing peak.

Coronal sections showing the electrode tracks in dorsal thalamus for the three rats can be seen in Fig. 7*C*. Tracks in rats 568 and 569 were clearly in ADN; 574 was a little more posterior and may possibly have been in lateral dorsal thalamus, from which HD cells have also been reported (Mizumori and Williams 1993). Recordings were made on East and West walls of the cuboid apparatus, which rats reached mainly via a pitch, but occasionally a mixed pitch/roll rotation from the adjacent South wall (Fig. 7*B*). Nineteen HD cells were recorded in 15 sessions from three rats (10, 3, and 2 sessions, respectively). HD cells were identified by their directional firing in baseline trials in a horizontal arena (Fig. 8); all were active on at least one wall and 15 were directionally modulated on both walls (Fig. 9). The other four cells (cells 2, 4, 5, and 9) were directionally modulated on one of the two walls but were not active on the opposing wall (defined as a rate less than 20% of the baseline rate); inspection of the dwell time distributions (thin lines in Fig. 9 plots) revealed that the animals undersampled the direction the cells might be expected to fire, given the dual-axis rule (i.e., downward, a direction that the animals tended to avoid), which may explain the lack of firing. The data below are reported as means ± SE. Facing directions (dwell times) on the East and West walls were inhomogeneously distributed but did not have a consistent relationship to the firing peaks on the East vs. West walls (Fig. 9).

Peak firing rates were significantly higher on baseline trials than on climbing trials (East and West walls averaged), averaging 28.16 ± 5.66 Hz on the flat and 20.52 ± 4.93 Hz on the walls [paired two-tailed *t*-test; *t*(14) = 3.29, *P* = 0.005]. General directionality of firing, expressed as Rayleigh vector scores, averaged 0.78 ±0.03 in baseline trials and 0.71 ± 0.04 in climbing trials; although slightly lower on the walls, these values did not significantly differ [paired two-tailed *t*-test; *t*(14) = 1.90, *P* = 0.08]. Tuning curve width, defined as the angular standard deviation of firing, averaged 0.64 ± 0.04° on baseline trials and 0.72 ± 0.05° in climbing trials; these values did not differ [*t*(14) = −1.74, *P* = 0.10]. These statistics suggest that HD cell firing is equally well directionally tuned, and thus as well modulated by yaw rotations during movement on the vertical plane as on the horizontal.

**Fig. 10.**
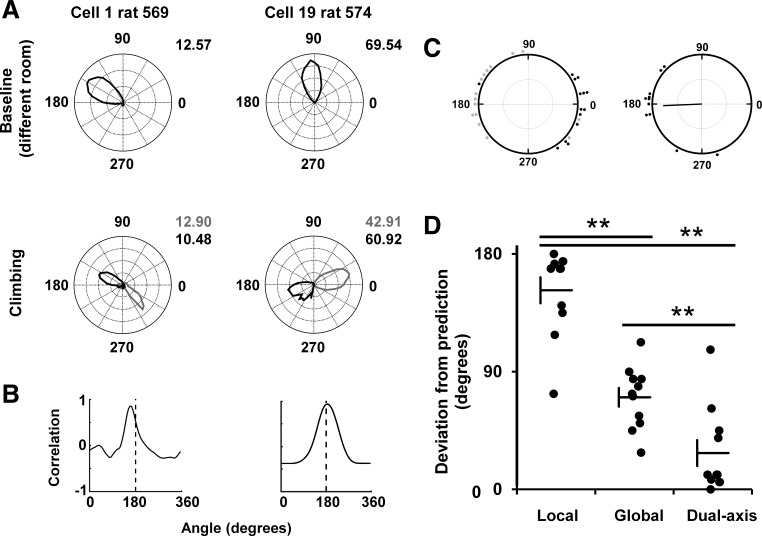
Properties of head direction cells recorded on the tree-trunk maze. *A*: polar plots of two cells, in baseline (top row) and tree-trunk (bottom row) trials, showing firing rate as a function of head direction. The tree-trunk trials are divided into East-wall (black) and West-wall (gray) epochs, with (viewed from the camera’s perspective) 90° being “up” and zero° being “right” etc. Firing rates at the peak direction are shown in black for the baseline and gray and black for West and East values, respectively. Note that the firing directions are 180° opposed. *B*: calculation of separation between East and West polar plots; the plots from each wall are cross-correlated (black line), with the peak being the amount of rotation that produces the highest correlation. This is close to 180° for both cells. *C*: *left*, distribution of firing directions of all 19 cells on East (black) and West (gray) walls. 0 = right; 90 = up; 180 = left; 270 = down. *Right*, circular plot of the angular difference (measured as the value of the peak cross-correlation) between firing on the two walls, for the cells that fired on both walls (all but four). Directions as for the left-hand plot. Each dot represents the value calculated for one cell, based on the cross-correlation method (see experimental procedures). The black line represents the mean direction for all cells. Note the clustering around 180° angular difference. *D*: comparison of the actual firing directions on the West wall against the value predicted by each of the three hypothetical models (data are jittered in the *x*-axis for ease of visualization). The dual-axis predictions were significantly different from both of the other models (**P < 0.01).

We then looked at the actual directions of firing. The firing of two example cells is shown in Fig. 10*A*, in which it can be seen that firing directions in each local, wall-anchored reference frame were ~180° apart on the East and West walls, a rotation that was quantified by finding the peak of a cross-correlation between the two plots (Fig. 10*B*). This ~180° offset was a pattern that was observed in all 15 sessions from all three animals; firing direction angular separations, calculated by the rotational autocorrelation procedure, averaged 182.42 ± 11.42° (Fig. 10*C*). These values were highly clustered (*R* = 0.78); a circular V-test confirmed that this was significantly concentrated around 180° [V = 8.59, *P* = 1.25 × 10^−4^]. We then compared the firing directions on the West wall with predictions made by transforming the East-wall firing direction according to each of the three models. To quantify these observations, we found the prediction error for each model, i.e., the difference of each data point from its predicted value according to the model. Errors were greatest for the local model (152.22 ± 9.92), and also higher for the global model (70.40 ± 7.17) than the dual-axis models (27.78 ± 9.92; Fig. 10*D*). A one-way ANOVA found a significant effect of model type [F(2,30) = 48.29, *P* = 0.0001]. Post hoc comparisons (Bonferroni corrected to alpha = 0.017) found a significant difference between local and global models [*t*(20) = 6.69, *P* = 1.66 × 10^−6^], the local and dual-axis models [*t*(20) = 8.89, *P* = 2.30 × 10^−8^], and the global and dual-axis models [*t*(20) = 3.48, *P* = 0.002]. Thus, the data are better fitted by the dual-axis model than either of the other two.

For one trial, one of the cameras was moved and an additional recording was made on the South wall; this showed a firing direction intermediate between the other two walls (Fig. 9 cell 1), lying to the left (counterclockwise) of the East wall pattern and to the right (clockwise) of the West wall pattern.

The data above are thus consistent with a dual-axis rotation rule.

## DISCUSSION

We have suggested that the activity of HD cells as animals move over a 3D surface may be organized by a dual-axis updating rule, in which firing directions are updated not just by yaw rotations about the animal’s D-V axis but also rotations of the D-V axis around the Earth-referenced (gravity-aligned) vertical axis. This rule allows generation of multiple planar local directional reference frames that can be linked together in global 3D space without inducing conflicts.

The issue of conflicts arising in directional reference frames for animals moving in 3D was first raised by Knierim et al. (2000), who found evidence that place cells recorded in microgravity are somehow able to avoid these conflicts and suggested that the local locomotor plane dominates encoding. Subsequently, Stackman et al. (2000) and Taube et al. (2013) showed that the firing of HD cells on a vertical surface is much like that of the same cells on a horizontal surface, as if the cells define the plane of locomotion to be the horizontal plane. These authors proposed a hemitorus model in which the cells’ activity could be accounted for by a pitch rotation of the floor’s horizontal coordinate system to any angle between ± 90°. However, the results of Stackman et al. and Taube et al. could be explained if the head direction system simply does not detect the transition of the rat from floor to wall; that is, it is insensitive to nonyaw rotations altogether, so that a cell that was firing on the floor, as the animal faced the wall, would simply keep firing as the animal climbed onto the wall. This insensitivity would lead to problems if the animal moved directly from one nonhorizontal surface to another, as shown in Fig. 3*A*. To avoid this problem, an additional rule suggests itself: not only should the system passively transfer its representation from the floor to a facing wall, but if the rat should directly move from one wall to another differently oriented one, the system should actively rotate the HD cell activity accordingly. Preservation of firing direction between the floor and the wall, as in Stackman et al. (2000) and Taube et al. (2013), can be accounted for by the fact that this rotation, around a horizontal axis, has no component around the Earth-vertical axis and thus invokes no HD network rotation.

With our modeling, we have confirmed that the dual-axis updating rule allows activity to be moved smoothly around a ring attractor (the usual architecture for modeling HD neurons (Redish et al. 1996; Skaggs et al. 1995; Zhang 1996) without discontinuities or conflicts (note that we restricted analysis to cases where the animal remained always with its head above its feet: the case of inversion is discussed further below) and it generalizes from a cuboid to free movement over a hemisphere, both concave and convex. The latter observation implies that an animal flying or swimming through volumetric space, which can be thought of as navigating over an invisible highly undulating surface, could also use this rule, with its attendant advantages of simplicity and economy.

Our data, showing appropriate rotation of HD cell firing for rats moving on opposing vertical walls, provide experimental support for the dual-axis rule. Note that under the dual-axis rule, “up” on one wall becomes “down” on the other — this conforms to an intuitive sense that the cell is “trying” to point (say) North. In this way, the system has found a simple way to compute a reasonable approximation to azimuth even though encoding azimuth is formally impossible on a vertical surface. Thus, not only does the rule avoid conflicts, it also provides a relatively straightforward link to global azimuth, which as mentioned in the introduction is the most navigationally relevant reference frame. In our experiment, the dual-axis rule also accounts for the behavior of four cells that switched off on one of the walls: this can be explained by noting that the rule would predict firing in a direction the rats undersampled (Fig. 9, cells 2, 4, 5, and 9).

Our analyses, both experimental and theoretical, were restricted to movement of an upright animal: what if the animal becomes inverted? We avoided modeling this scenario because on inversion the D-V axis of the rat, which is central to the dual-axis rule, reverses through zero (i.e., horizontal — in which azimuth, itself being referenced to the horizontal plane, is therefore undefined). It thus moves from positive (dorsal up) to negative (ventral up), which would have necessitated a sudden jump of the network state from one “side” of the ring attractor to the other. This singularity in the dual-axis model offers a potential explanation for previous observations that in rats, a pitch (head-over-heels) rotation to inversion causes HD firing to degrade (Calton and Taube 2005) and navigational efficacy to degrade (Valerio et al. 2010). In bats, azimuth reverses instead, permitting the network state to remain unaltered (i.e., cells keep firing; Finkelstein et al. 2015; see Jeffery et al. 2015 for more detailed discussion of this issue).

Our investigations were conducted in rats and only pertain to movement over a surface; different issues arise for animals that can move freely through a volumetric space, as bats, birds, and fish do. In bats, which are mammals that can move freely in volumetric space, it appears that HD cells have tuning curves specific to pitch angle (unlike rats in which pitch-sensitive cells appear modulated by, rather than tuned to, pitch (Stackman and Taube 1998), and many cells show conjunctive encoding suggestive of a more volumetric encoding scheme than the planar one evident here (Finkelstein et al. 2015). However, the population of azimuth-specific cells far exceeds those specific to roll or pitch, suggesting that even in bats the encoding has a planar bias, although the dominant plane may be the Earth-horizontal rather than the locomotor surface as appears to be the case with rats.

The dual-axis rule combines egocentric and allocentric reference frames — egocentric from rotation of the animal around its own D-V axis, and allocentric from rotation of the D-V axis around gravity — and thus allows a directional reference frame to be always self-consistent in global space. The idea that gravity might serve as an organizing framework for representing allocentric space was put forward by Yakusheva et al. (2007) and gains support from studies showing a role for the vestibuloceberebellum and thalamus in processing rotations in 3D space. Horizontal rotation is detected with the labyrinthine semicircular canals (Angelaki and Cullen 2008; Brown 1874), but gravity, which is a form of linear acceleration, is detected by the saccule in the otolith organs. In support of this notion, otoconia-deficient mice, with disrupted otolith function, show impaired HD cell encoding (Yoder and Taube 2009), and also navigation deficits (Yoder and Kirby 2014). Angelaki and colleagues have shown that neurons in the vestibular nuclei that receive otolith afferents have response properties that would contribute determination of the gravity vector relative to off-axis rotation of the head (Angelaki 1992a, 1992b). Another structure likely to be involved is the vestibulocerebellum, downstream of the vestibular nuclei, where neurons extract the gravity vector from a combination of otolith and semicircular canal signals (Angelaki and Hess 1995). There are abundant projections from here to the head direction circuit (see Shinder and Taube 2010 for review). Most recently, Angelaki and colleagues have reported gravity-tuned HD cells in the anterior thalamus of macaques (Laurens et al. 2016), suggesting a possible entry point for gravity to reach the head direction system.

In summary, the dual-axis rule allows a consistent representation of head direction even over complex undulating surfaces, such that local representations of head direction are linked by their relations with respect to the gravity-defined vertical axis. In this way, a stable sense of direction can be maintained at any point, without errors or conflicts, allowing animals to remain oriented as they navigate over complex terrain.

## NOTE ADDED IN PROOF

During publication of this article we became aware of new results from the Taube group that support the dual-axis, or “mosaic,” model. Dumont et al. (2017) recorded HD cells from the ADN while rats traveled different paths to reach the top surface of a cube and found consistent HD cell firing orientation on the top surface, regardless of the path the animal took to get there. These findings provide additional support for the notion that the system can prevent Berry phase errors by accounting for nonhorizontal rotations.

## GRANTS

This work was supported by BBSRC (BB/J009792/1), MRC (G1100669), and Wellcome (no. 083540) grants to K. J. Jeffery and a BBSRC CASE studentship (BB/F015968/1) to J. J. Wilson in collaboration with Axona Ltd.

## DISCLOSURES

No conflicts of interest, financial or otherwise, are declared by the authors. K. J. Jeffery is a nonshareholding director of Axona Ltd.

## AUTHOR CONTRIBUTIONS

H.J.I.P., J.J.W., and K.J.J. conceived and designed research; H.J.I.P. and J.J.W. performed experiments; H.J.I.P., J.J.W., and K.J.J. analyzed data; H.J.I.P., J.J.W., and K.J.J. interpreted results of experiments; H.J.I.P., J.J.W., and K.J.J. edited and revised manuscript; H.J.I.P., J.J.W., and K.J.J. approved final version of manuscript; H.J.I.P., J.J.W., and K.J.J. prepared figures; K.J.J. drafted manuscript.

## APPENDIX

### Model Equations

The activation level $h_{i}^{HD}\left(t\right)$ of head direction cell *i* at time *t* is given by

$$\tau^{HD}\frac{dh_{i}^{HD}\left(t\right)}{dt}=e_{i}^{INIT}\left(t\right)+e_{i}^{AHV+G}\left(t\right)+\frac{\phi_{1}}{C^{HD\to HD}}\underset{j}{\sum }w_{ij}r_{j}^{HD}(t-\text{Δ}t)-\frac{1}{N^{HD}}\underset{j}{\sum }\omega^{HD}r_{j}^{HD}\left(t\right)$$

where $h_{i}^{HD}\left(t\right)$ is the activation level of HD cell *i* at time *t*. The term τ^HD^ is the neuronal time constant. The term $e_{i}^{INIT}\left(t\right)$ reflects external input to the network and is used to initialize an activity packet at the beginning of simulation. It is determined by a Gaussian function, described in the simulation protocol.

The term $\frac{\phi_{1}}{C^{HD\to HD}}\underset{j}{\sum }w_{ij}r_{j}^{HD}(t-\text{Δ}t)$ reflects excitatory influence of HD cells upon one another, where *w_ij_* is the synaptic weight from presynaptic HD cell *j* onto postsynaptic HD cell *i* and $r_{j}^{HD}\left(t-\text{Δ}t\right)$ is the firing rate of presynaptic HD cell *j* Δ*t* in the past, as according to a naturally occurring synaptic delay. ϕ_1_ is a parameter controlling the overall strength of recurrent synaptic input within the HD layer, scaled by the number of synapses each postsynaptic HD cell receives (C^HD⃗HD^).

The term $\frac{1}{N^{HD}}\underset{j}{\sum }\omega^{HD}r_{j}^{HD}\left(t\right)$ describes inhibition within the HD layer. The term ω^HD^ is a global inhibition parameter, while *N*^HD^ is the total number of HD cells in the layer. In this manner, constant inhibition is applied to all HD cells in a time-varying manner based on overall network activity. While this trades some realism by not modeling interneurons, it provides a simple mechanism to provide the inhibition required for a CANN.

The term $e_{i}^{AHV+G}\left(t\right)$ reflects self-motion input that is generated by the combined influence of rotations about the D-V axis and rotations of the D-V axis about the gravity vector.

The firing rate $r_{i}^{HD}\left(t\right)$ of HD cell *i* at time *t* was calculated using the sigmoid transfer function

$$r_{i}^{HD}\left(t\right)=\frac{1}{1+e^{-2\beta \left[h_{i}^{HD}\left(t\right)-\alpha \right]}}$$

where α and β and are the sigmoid threshold and slope, respectively. As this is a firing-rate model, individual action potentials are not simulated, and neuronal activity is represented as this instantaneous firing rate.

Recurrent weights within the HD layer were prewired using the Gaussian function

$$w_{ij}=e^{-{\left(s_{ij}^{RC}\right)}^{2}/2{\left(\sigma^{RC}\right)}^{2}}$$

where *w_ij_* is the weight from presynaptic HD cell *j* onto postsynaptic HD cell *i* and σ^RC^ is the standard deviation of the Gaussian profile. $s_{ij}^{RC}$ is the difference between the preferred firing directions *x_i_* and *x_j_* of the post- and presynaptic HD cells *i* and *j*, respectively. It is calculated as

$$S_{ij}^{RC}=MIN\left(\left|x_{i}-x_{j}\right|,360-\left|x_{i}-x_{j}\right|\right)$$

which allows weights between HD cells to be continuous around the attractor ring across the 0/360° divide. The weight profile is normalized after initialization, by ensuring that the square root of the sum of the squares of receiving weights for a given postsynaptic HD cell *i* is limited to 1. This is calculated as

$$\sqrt{\underset{j}{\sum }{\left(w_{ij}\right)}^{2}}=1$$

The differential equations for this model were not solved analytically but were implemented by making discrete approximations of their solutions using a Forward Euler finite difference scheme with a timestep size of δ*t*.

### Simulation Protocol

At the beginning of each simulation, firing rates and activations of all HD cells were set to zero. Recurrent collateral connections were prewired and synaptic weights normalized. Static external input $e_{i}^{INIT}$ was applied for 100 ms, and both HD activations and firing rates updated as described above. External input was calculated for each HD cell *i* by the Gaussian profile

$$e_{i}^{INIT}=\lambda^{INIT}e^{-{\left(s_{i}^{INIT}\right)}^{2}/2{\left(\sigma^{\;INIT}\right)}^{2}}$$

where λ^INIT^ is a scaling factor determining the overall strength of this input, and σ ^INIT^ is the Gaussian standard deviation. $s_{i}^{INIT}$ is the difference between the location of this input (i.e., the true head direction of the rat at the beginning of simulation) *x*^INIT^ and the preferred firing direction *x_i_* of postsynaptic HD cell *i*. It is calculated as

$$S_{i}^{INIT}=MIN\left(\left|x_{i}-x^{INIT}\right|,360-\left|x_{i}-x^{INIT}\right|\right)$$

which, as with prewiring of recurrent weights, creates a wrap-around effect over the 360/0° divide. This input acts solely to initialize an activity packet at location *x*^INIT^.

Simulated paths were generated with custom-built MATLAB scripts, producing a random walk over the surface of either a hemisphere or a cuboid apparatus (the latter in direct replication of the experimental scenario). After 100 ms, external initializing input to the network was removed and the network simulated for a further 600 s. HD firing rates and activations are updated as described above. The network was then fed with a set of 6,000 pairs of [*x y z*] vectors from the path, describing the position

$$\overset{⃗}{pos}=\left[x_{pos},y_{pos},z_{pos}\right]$$

and allocentric head direction

$$\overset{⃗}{head}=\left[x_{head},y_{head},z_{head}\right]$$

of the rat at 100-ms intervals.

Self-motion-based update to the network is represented by a Gaussian input to the HD layer. The location of this Gaussian input is offset from the current activity packet location by an increment which is the sum of the change in heading within the current locomotor plane, and the amount by which the current plane has rotated about the vertical axis of gravity.

During the 600 s of random walk, the input term $e_{i}^{AHV+G}$ is updated. It is determined by Gaussian profile

$$e_{i}^{AHV+G}=\lambda^{AHV+G}e^{-{\left(s_{i}^{AHV+G}\right)}^{2}/2{\left(\sigma^{\;AHV+G}\right)}^{2}}$$

where λ^AHV+G^ is a scaling factor determining input strength, σ^AHV+G^ is the Gaussian standard deviation, and $s_{i}^{AHV+G}$ is the difference between this input location *x*^AHV+G^ and the preferred firing direction *x_i_* of postsynaptic HD cell *i*. It is calculated as

$$S_{i}^{AHV+G}=MIN\left(\left|x_{i}-x^{AHV+G}\right|,360-\left|x_{i}-x^{AHV+G}\right|\right)$$

which, again, ensures a wrap-around effect. The location of this input *x*^AHV+G^ is set to the current HD of the rat at the beginning of simulation and is then updated dynamically as the rat’s location and heading in 3D space changes. At each path step after the first, it is updated by the sum of the increments *I*^AHV^ and *I*^G^, which reflect rotations about the D-V axis, and rotations of the D-V axis about the gravity vector, respectively. That is

$$x^{AHV+G}\left(t+1\right)=x^{AHV+G}\left(t\right)+I^{AHV}\left(t\right)+I^{G}\left(t\right)$$

In this manner, self-motion input updates the HD representation to reflect both sorts of rotation.

In “no gravity” simulations, *I*^G^ is artificially set to and kept at 0.

### Calculation of Self-Motion-Based Update Components

Table 1 shows parameters for all simulations (hemisphere and cuboid, in both gravity-rule and no-gravity-rule conditions). As described in the introduction, to update its heading estimate the model computes two separate self-motion components: head rotations relative to the D-V axis and D-V axis rotations relative to the vertical axis.

**Table 1.** Parameters for all simulations (hemisphere and cuboid, in both gravity-rule and no-gravity-rule conditions)

Parameter	Value
Simulation time	600.1 s
Input time	0.1 s
Path time	600 s
Head direction cells	500
δ*t*	0.001 s
RC connections	500
ϕ_1_	1
σ^RC^	20°
Δ*t*	0.005 s
τ^HD^	0.01 s
α	0
β	0.3
ω^HD^	0.2
λ^INIT^	20
χ^INIT^	90°
σ^INIT^	20°
λ^AHV+G^	50
σ^AHV+G^	30°

### Head Rotations in the Current Locomotor Plane

Head rotations in the current locomotor plane (i.e., those which occur around the D-V axis of the rat) were calculated when the locomotor plane’s orientation with respect to gravity changed between timesteps. To do this, an equivalent reference vector is taken each timestep, and change in head direction is measured relative to this reference vector.

Rotation about the D-V axis (*I*^AHV^) would be biologically derived from the vestibular system. Computationally, it is calculated as the change in heading in relationship to a reference vector in the current plane (the current surface on the cuboid, or the tangent plane at the current position on the hemisphere). It is calculated as

$$I^{AHV}\left(t\right)=\alpha \left(t+1\right)-\alpha \left(t\right)$$

where α(*t*) gives the angle between the current heading and a reference vector at time (*t*), and α(*t*+1) gives the angle between the heading at time (*t*+1) and a reference vector at time (*t*+1). This is therefore a measure of head rotation about the D-V axis across timesteps. These angles were calculated, for a given timestep, as the arctangent of the norm of the cross product of the reference vector and the heading vector at that timestep, and the dot product of the reference vector and the heading vector

$$\alpha \left(t\right)=atan2\left[\left|\overset{⃗}{RV}\left(t\right)\times \overset{⃗}{head}\left(t\right)\right|,\overset{⃗}{RV}\left(t\right)\cdot \overset{⃗}{head}\left(t\right)\right]$$

where $\overset{⃗}{RV}\left(t\right)$ is the reference vector at time (*t*) (described in more detail below), and $\left|\overset{⃗}{RV}\left(t\right)\times \overset{⃗}{head}\left(t\right)\right|$ is the norm of the cross product between current reference vector and current heading, and $\overset{⃗}{RV}\left(t\right)\cdot \overset{⃗}{head}\left(t\right)$ is the dot product of the current reference vector and current heading.

The above formula does not account for handedness when measuring angle and so does not distinguish clockwise from counterclockwise rotations. The above formula was therefore signed by the following correcting formula

$$\alpha =\{\begin{array}{c} 2\pi -\alpha \\ \alpha \end{array}\begin{array}{c} if\;\overset{⃗}{SN}\cdot \left(\overset{⃗}{RV}\times \overset{⃗}{head}\right)<0\\ otherwise \end{array}$$

which both takes into account handedness and returns α in the range [0,2π].

The term $\overset{⃗}{SN}$ in the above formula is the unit vector describing surface normal to the plane on which the rat is currently located and thus corresponds to the current D-V axis of the rat. For paths across the hemisphere, it is calculated based on the fact that the simulated hemisphere is half of a unit sphere defined by

$$U\left(x,y,z\right)=x^{2}+y^{2}+z^{2}=1$$

The surface normal at $\overset{⃗}{pos}$ corresponds to the surface normal to the unit sphere at this point. The normal vector of a sphere at a given point is equal to the gradient at that point, such that position can be substituted into the formula for the gradient (i.e., the derivative) of the unit sphere, ∇*U*

∇U=(2x,2y,2z)

which allows substitution of position

$$\overset{⃗}{SN}=\nabla U\overset{⃗}{pos}=\left(2x_{pos},2y_{pos},2z_{pos}\right)$$

The surface normal is converted to a unit vector by normalizing it

$$\overset{⃗}{SN}=\frac{\overset{⃗}{SN}}{\left|\overset{⃗}{SN}\right|}$$

For cuboid simulations, the surface normal is simply taken as an orthogonal vector to the current flat surface on which the rat is currently positioned.

The reference vector $\overset{⃗}{RV}$ is the direction from the current position to the point of intersection of the *z* axis and the current tangent plane. The reference vector therefore always points from the current position toward the vertical axis defined by gravity, which runs through the middle of the cuboid or hemisphere. It is calculated in hemisphere simulations by first finding the point in the current tangent plane where *z* = 1, and *x* and *y* are defined by $\overset{⃗}{pos}$. This is done by defining the current tangent plane in terms of $\vec{SN}$ and $\overset{⃗}{pos}$ as

$$x_{SN}\left(x-x_{pos}\right)+y_{SN}\left(y-y_{pos}\right)+z_{SN}\left(z-z_{pos}\right)=0$$

Any point that lies along the reference vector between the current position and the intersection of the *z* axis and the current tangent plane can be found by using the fact that, looking from directly above and considering only the *x,y* plane, any pair of [*x*,*y*] values between the current position and the *z* axis must lie along the line defined by $\overset{⃗}{pos}$. This fact allows redefinition of *y* in terms of *x* as follows

$$y=\frac{pos_{x}}{pos_{y}}x$$

This formula can be substituted back into the tangent plane equation along with *z* = 1 to solve for *x*, which itself can be substituted back into the tangent plane equation along with the derived value of *x* to find *y*. This therefore yields an [*x*,*y*,*z*] coordinate, referred to as $\overset{⃗}{\mu pos}$ of a position on the current tangent plane that lies in the direction of the vector from $\overset{⃗}{pos}$ to the point of intersection of the *z* axis and the current plane. The reference vector is then taken as the normalized direction to this new position from $\overset{⃗}{pos}$

$$\overset{⃗}{RV}=\frac{\overset{⃗}{\mu pos}-\overset{⃗}{pos}}{\left|\overset{⃗}{\mu pos}-\overset{⃗}{pos}\right|}$$

For cuboid simulations, $\overset{⃗}{RV}$ is set, on all vertical walls as [0,0,1] (i.e., pointing directly upward). For all situations in which the current tangent plane and the tangent plane at the next timestep is parallel to the [*x,y*] plane (i.e., parallel to the earth horizontal), $\overset{⃗}{RV}$ is set as North (defined as [0,1,0] in these simulations). This is particularly important when the rat is navigating on the top of the cuboid, as a fixed reference vector is required to accurately compute rotations about the D-V axis. When transitioning between any of the vertical walls and the top surface, $\overset{⃗}{RV}$ during the timestep on which the rat is positioned on the top face of the cuboid (i.e., when the rat is just about to move from the top to a wall, or has just moved from a wall to the top) is calculated as the direction toward the *z*-axis by using the above formula with $\overset{⃗}{\mu pos}$ = [0,0,1]. This allows a consistent reference vector when transitioning from vertical walls to the top surface of the cuboid.

### Rotation of Locomotor Frame About Axis of Gravity

Rotations of the current reference frame about gravity are calculated as rotation of the D-V axis around the vertical axis defined by gravity. This is calculated as the change in azimuth angle of the surface normal (i.e., the D-V axis of the rat) between timesteps. These rotations are added to those performed about the D-V axis, and together these two sorts of rotation influence HD activity packet location.

Rotations of the D-V axis about the central axis of gravity, reflected by *I^G^* are calculated as the change in azimuth angle of $\vec{SN}$ between positions

$$I^{G}=atan2\left\{\text{sin}\left[\theta \left(t+1\right)-\theta (t)]\},\{[\text{cos}\theta \left(t+1\right)-\theta \left(t\right)\right]\right\}$$

which yields the signed angle from θ(*t*) to θ(*t*+1), where θ(*t*) is the azimuth angle of $\vec{SN}$ at time (*t*), calculated as

$$\theta \left(t\right)=atan2\left[y_{SN}\left(t\right),x_{SN}\left(t\right)\right]$$

It should be noted that, while atan2{sin[θ(*t* + 1) − θ(*t*)]}, {cos[θ(*t* + 1) −θ(*t*)]} yields the answer of {cos[θ(*t* +1 ) − θ(*t*)]}, this answer will, unlike a simple subtraction, always give the correct signed difference between θ(*t* + 1) and θ(*t*) even when these two quantities are on different sides of the 0/360 divide. As with calculations of *I*^AHV^, corrections must be made for transitions between cuboid top and walls. When $\vec{SN}$ points directly upward, as it will on top cuboid face, θ(*t*) = 0 and movements onto the walls will cause a false change in *I*^G^. Therefore, *I*^G^ is set to zero when making these transitions, as no true gravity rotations have occurred.

### Model Analysis

HD firing rate data were analyzed by taking the direction indicated by current HD packet location. This is calculated, based on the known cell preferred firing directions, as a population vector statistic

$$PV\left(t\right)=arctan\left[\frac{\sum_{i}r_{i}\text{sin}\left(PD_{i}\right)}{\sum_{i}r_{i}\text{cos}\left(PD_{i}\right)}\right]$$

where PD*_i_* is the preferred firing direction of HD cell *i* with firing rate *r_i_*. When using this arctan-based formula on this population vector, return values are bound in the range [−π/2, π/2] and are signed based on the quadrant: they will be positive in quadrants I and III and negative in quadrants II and IV.

The following formula will correct the angle returned by arctan so that it lies in the range [0, 2π]. For the sake of readability, $\underset{i}{\sum }r_{i}\text{sin}\left(PD_{i}\right)$ is substituted for *y* and $\underset{i}{\sum }r_{i}\text{cos}\left(PD_{i}\right)$ is substituted for *x*

$$PV\left(t\right)=\{\begin{array}{c} \arctan\left(\frac{y}{x}\right)ify>0,x>0\\ \arctan\left(\frac{y}{x}\right)+\pi if\;x<0\\ \arctan\left(\frac{y}{x}\right)+2\pi if\;x>0,y<0 \end{array}$$

which gives the overall direction signaled by the HD layer. This direction is compared with the direction of the current heading, once rotated back into the [*x*,*y*] horizontal plane (rotations performed using the MATLAB function *rodrigues_rotation.m*, downloaded from the file exchange), which is given as

$$dir=\{\begin{array}{c} 2\pi +a\;tan2(y_{ROT},x_{ROT})\;if\;atan2\left(y_{ROT},x_{ROT}\right)<0\\ atan2(y_{ROT},x_{ROT})\;otherwise \end{array}$$

where *ROT* denotes *x* and *y* coordinates of rotated headings. The direction indicated by the cell which previously had a preferred direction of North is then calculated as

North cell direction=PV−dir

This analysis is performed every 100 ms, before the next set of heading and position vectors are fed into the network.

The tuning curve for a given cell within a single wall was calculated by binning current heading within the wall frame of reference into 6° bins and averaging firing rate across each bin. This was then plotted against directional heading with the wall frame of reference. As with the experimental data, Gaussian smoothing with a 5° kernel was used. Heading within the wall frame of reference was calculated by taking the angle between the upward-facing vector (0,0,1) and the current heading vector, given by the formula

$$\overset{⃗}{head_{W}}=\text{acos}\left[\overset{⃗}{head}\cdot \left(0,0,1\right)\right]$$

This angle was signed according to the following formula

$$\overset{⃗}{head_{W}}=\{\begin{array}{c} 2\pi -\overset{⃗}{head_{W}}\;if\;\overset{⃗}{Compass}\cdot \left[\overset{⃗}{head}\times \left(0,0,1\right)\right]<0\\ \overset{⃗}{head_{W}}\;otherwise \end{array}$$

where $\vec{Compass}$ is a wall-dependent vector representing the compass direction of the current wall. For the North wall, it is (0,1,0), for South it is (0,−1,0), for East it is (1,0,0), and for West it is (−1,0,0). This analysis is also performed every 100 ms.

## References

[B1] Angelaki DE Detection of rotating gravity signals. Biol Cybern 67: 523–533, 1992 a. doi:10.1007/BF00198759. 1472576

[B2] Angelaki DE Two-dimensional coding of linear acceleration and the angular velocity sensitivity of the otolith system. Biol Cybern 67: 511–521, 1992 b. doi:10.1007/BF00198758. 1472575

[B3] Angelaki DE, Cullen KE Vestibular system: the many facets of a multimodal sense. Annu Rev Neurosci 31: 125–150, 2008. doi:10.1146/annurev.neuro.31.060407.125555. 18338968

[B4] Angelaki DE, Hess BJ Inertial representation of angular motion in the vestibular system of rhesus monkeys. II. Otolith-controlled transformation that depends on an intact cerebellar nodulus. J Neurophysiol 73: 1729–1751, 1995. 7623076 10.1152/jn.1995.73.5.1729

[B5] Brown AC The sense of rotation and the anatomy and physiology of the semicircular canals of the internal ear. J Anat Physiol 8: 327–331, 1874. 17231027 PMC1318976

[B6] Calton JL, Taube JS Degradation of head direction cell activity during inverted locomotion. J Neurosci 25: 2420–2428, 2005. doi:10.1523/JNEUROSCI.3511-04.2005. 15745969 PMC6726092

[B7] Calton JL, Turner CS, Cyrenne D-LM, Lee BR, Taube JS Landmark control and updating of self-movement cues are largely maintained in head direction cells after lesions of the posterior parietal cortex. Behav Neurosci 122: 827–840, 2008. doi:10.1037/0735-7044.122.4.827. 18729636 PMC2771080

[B8] Clark BJ, Harris MJ, Taube JS Control of anterodorsal thalamic head direction cells by environmental boundaries: comparison with conflicting distal landmarks. Hippocampus 22: 172–187, 2012. doi:10.1002/hipo.20880. 21080407

[B9a] Dumont JR, Lachance PA, Marcroft JL, Bovio NR, Winter SS, Taube JS Are head direction cell responses commutative on a 3D surface? Society for Neuroscience abstracts, 427.11, 2017 http://www.abstractsonline.com/pp8/index.html#!/4376/presentation/5317.

[B9] Finkelstein A, Derdikman D, Rubin A, Foerster JN, Las L, Ulanovsky N Three-dimensional head-direction coding in the bat brain. Nature 517: 159–164, 2015. doi:10.1038/nature14031. 25470055

[B10] Jeffery KJ, Wilson JJ, Casali G, Hayman RM Neural encoding of large-scale three-dimensional space-properties and constraints. Front Psychol 6: 927, 2015. doi:10.3389/fpsyg.2015.00927. 26236246 PMC4501222

[B11] Knierim JJ, McNaughton BL, Poe GR Three-dimensional spatial selectivity of hippocampal neurons during space flight. Nat Neurosci 3: 209–210, 2000. doi:10.1038/72910. 10700250

[B12] Knierim JJ, Zhang K Attractor dynamics of spatially correlated neural activity in the limbic system. Annu Rev Neurosci 35: 267–285, 2012. doi:10.1146/annurev-neuro-062111-150351. 22462545 PMC5613981

[B13] Laurens J, Kim B, Dickman JD, Angelaki DE Gravity orientation tuning in macaque anterior thalamus. Nat Neurosci 19: 1566–1568, 2016. doi:10.1038/nn.4423. 27775722 PMC5791896

[B14] Mizumori SJ, Williams JD Directionally selective mnemonic properties of neurons in the lateral dorsal nucleus of the thalamus of rats. J Neurosci 13: 4015–4028, 1993. 8366357 10.1523/JNEUROSCI.13-09-04015.1993 PMC6576470

[B15] Paxinos G, Watson C The Rat Brain in Stereotaxic Coordinates (6th ed.). London: Academic, 2007.

[B16] Redish A, Elga A, Touretzky D A coupled attractor model of the rodent head direction system. Netw Comput Neural Syst 7: 671–685, 1996. doi:10.1088/0954-898X_7_4_004.

[B17] Shinder ME, Taube JS Differentiating ascending vestibular pathways to the cortex involved in spatial cognition. J Vestib Res 20: 3–23, 2010. 20555163 10.3233/VES-2010-0344

[B18] Skaggs WE, Knierim JJ, Kudrimoti HS, McNaughton BL A model of the neural basis of the rat’s sense of direction. In: Advances in Neural Information Processing Systems 7, edited by Tesauro G, Touretzky DS, Leen TK Cambridge, MA: MIT Press, 1995, p. 173–180. 11539168

[B19] Stackman RW, Taube JS Firing properties of rat lateral mammillary single units: head direction, head pitch, and angular head velocity. J Neurosci 18: 9020–9037, 1998. 9787007 10.1523/JNEUROSCI.18-21-09020.1998 PMC1550347

[B20] Stackman RW, Tullman ML, Taube JS Maintenance of rat head direction cell firing during locomotion in the vertical plane. J Neurophysiol 83: 393–405, 2000. 10634882 10.1152/jn.2000.83.1.393

[B21] Stringer SM, Trappenberg TP, Rolls ET, de Araujo IET Self-organizing continuous attractor networks and path integration: one-dimensional models of head direction cells. Network 13: 217–242, 2002. doi:10.1080/net.13.2.217.242. 12061421

[B22a] Swanson LW Brain Maps: Structure of the Rat Brain (3rd ed.). Amsterdam: Elsevier, 2004 http://larrywswanson.com/?page_id=164.

[B22] Taube JS The head direction signal: origins and sensory-motor integration. Annu Rev Neurosci 30: 181–207, 2007. doi:10.1146/annurev.neuro.29.051605.112854. 17341158

[B23] Taube JS, Muller RU, Ranck JB Jr Head-direction cells recorded from the postsubiculum in freely moving rats. I. Description and quantitative analysis. J Neurosci 10: 420–435, 1990 a. 2303851 10.1523/JNEUROSCI.10-02-00420.1990 PMC6570151

[B24] Taube JS, Muller RU, Ranck JBJ Jr Head-direction cells recorded from the postsubiculum in freely moving rats. II. Effects of environmental manipulations. J Neurosci 10: 436–447, 1990 b. 2303852 10.1523/JNEUROSCI.10-02-00436.1990 PMC6570161

[B26] Taube JS, Wang SS, Kim SY, Frohardt RJ Updating of the spatial reference frame of head direction cells in response to locomotion in the vertical plane. J Neurophysiol 109: 873–888, 2013. doi:10.1152/jn.00239.2012. 23114216 PMC3567391

[B27] Tsanov M, Chah E, Vann SD, Reilly RB, Erichsen JT, Aggleton JP, O’Mara SM Theta-modulated head direction cells in the rat anterior thalamus. J Neurosci 31: 9489–9502, 2011. doi:10.1523/JNEUROSCI.0353-11.2011. 21715614 PMC3855197

[B28] Valerio S, Clark BJ, Chan JHM, Frost CP, Harris MJ, Taube JS Directional learning, but no spatial mapping by rats performing a navigational task in an inverted orientation. Neurobiol Learn Mem 93: 495–505, 2010. doi:10.1016/j.nlm.2010.01.007. 20109566 PMC2862784

[B25] Wilson J, Page HJI, Jeffery KJ A proposed rule for updating of the head direction cell reference frame following rotations in three dimensions. bioRxiv 2016. doi:10.1101/043711. PMC5866468 29021391

[B29] Yakusheva TA, Shaikh AG, Green AM, Blazquez PM, Dickman JD, Angelaki DE Purkinje cells in posterior cerebellar vermis encode motion in an inertial reference frame. Neuron 54: 973–985, 2007. doi:10.1016/j.neuron.2007.06.003. 17582336

[B30] Yoder RM, Kirby SL Otoconia-deficient mice show selective spatial deficits. Hippocampus 24: 1169–1177, 2014. doi:10.1002/hipo.22300. 24802640 PMC4209000

[B31] Yoder RM, Taube JS Head direction cell activity in mice: robust directional signal depends on intact otolith organs. J Neurosci 29: 1061–1076, 2009. doi:10.1523/JNEUROSCI.1679-08.2009. 19176815 PMC2768409

[B32] Zhang K Representation of spatial orientation by the intrinsic dynamics of the head-direction cell ensemble: a theory. J Neurosci 16: 2112–2126, 1996. 8604055 10.1523/JNEUROSCI.16-06-02112.1996 PMC6578512

